# Novel Insights into the Distribution and Functional Aspects of the Calcium Binding Protein Secretagogin from Studies on Rat Brain and Primary Neuronal Cell Culture

**DOI:** 10.3389/fnmol.2012.00084

**Published:** 2012-08-06

**Authors:** Magdalena Maj, Ivan Milenkovic, Jan Bauer, Tord Berggård, Martina Veit, Aysegül Ilhan-Mutlu, Ludwig Wagner, Verena Tretter

**Affiliations:** ^1^Department of Internal Medicine III, Division of Nephrology and Dialysis, Medizinische Universität WienVienna, Austria; ^2^Department of Biochemistry and Molecular Biology, Center for Brain Research, Medizinische Universität WienVienna, Austria; ^3^Institute of Neurology, Medizinische Universität WienVienna, Austria; ^4^Department of Neuroimmunology, Center for Brain Research, Medizinische Universität WienVienna, Austria; ^5^Alligator BioscienceLund, Sweden

**Keywords:** Secretagogin, calcium binding proteins, neuronal cell marker, insulin

## Abstract

Secretagogin is a calcium binding protein (CBP) highly expressed in neuroendocrine cells. It has been shown to be involved in insulin secretion from pancreatic beta cells and is a strong candidate as a biomarker for endocrine tumors, stroke, and eventually psychiatric conditions. Secretagogin has been hypothesized to exert a neuroprotective role in neurodegenerative diseases like Alzheimer’s disease. The expression pattern of Secretagogin is not conserved from rodents to humans. We used brain tissue and primary neuronal cell cultures from rat to further characterize this CBP in rodents and to perform a few functional assays *in vitro*. Immunohistochemistry on rat brain slices revealed a high density of Secretagogin-positive cells in distinct brain regions. Secretagogin was found in the cytosol or associated with subcellular compartments. We tested primary neuronal cultures for their suitability as model systems to further investigate functional properties of Secretagogin. These cultures can easily be manipulated by treatment with drugs or by transfection with test constructs interfering with signaling cascades that might be linked to the cellular function of Secretagogin. We show that, like in pancreatic beta cells and insulinoma cell lines, also in neurons the expression level of Secretagogin is dependent on extracellular insulin and glucose. Further, we show also for rat brain neuronal tissue that Secretagogin interacts with the microtubule-associated protein Tau and that this interaction is dependent on Ca^2+^. Future studies should aim to study in further detail the molecular properties and function of Secretagogin in individual neuronal cell types, in particular the subcellular localization and trafficking of this protein and a possible active secretion by neurons.

## Introduction

Calcium (Ca^2+^) is a universal second messenger, which plays a crucial role in signal transduction in many fundamental physiological processes. In the central nervous system (CNS) calcium signaling is utilized by neurons to control membrane excitability, neurotransmitter release, gene expression, cellular growth, differentiation, and cell death. Different variants of high transient and local Ca^2+^ concentrations in neurons play an important role in information processing (Niggli and Shirokova, 2007). Intracellular Ca^2+^ waves are also observed in glia cells, a finding that currently reshapes the known role of glia cells from neuronal supporters to active signal transmitting cells (Dityatev and Rusakov, 2011).

Calcium binding proteins (CBPs) therefore play a pivotal role in sensing and transducing these signals into cellular responses like the modulation of ion channel or receptor function, enzyme activity, neurotransmitter release, and many more (Yanez et al., 2012). The huge number of identified CBPs relates to their different roles and substrate specificity. N-terminal myristoylation and specific protein–protein interactions mediate their subcellular targeting and substrate specificity. Calcium also plays an important role in the secretory pathway of neurons regulating processes like vesicle budding and fusion, vesicle trafficking between Golgi stacks, TGN sorting, and the regulation of SNARE proteins. Some CBPs like calmodulin are possibly present in all neuronal cell types. Other known neuronal CBPs (nCBPs) are present only in certain subpopulations of neurons (Mikhaylova et al., 2011). From the functional point of view two major groups of nCBPs have traditionally been distinguished: Ca^2+^ buffers, which bind Ca^2+^ with high affinity without undergoing major conformational changes, and Ca^2+^ sensors (NCS), which need higher Ca^2+^ pulses for activation and undergo substantial conformational changes upon binding to Ca^2+^. These activated states lead to the exposure of interaction motifs with downstream targets and frequently initiate signal transduction cascades (Burgoyne and Haynes, 2012). One of the best-known and well-studied examples also in this respect is again calmodulin. The distinction between calcium buffers and calcium sensors is still valid from a functional viewpoint, but it becomes increasingly difficult to assign individual CBPs selectively to one or the other group. The more experimental evidence is available, the more it becomes clear that CBPs might have dual or more functional properties (Schwaller, 2009; Mikhaylova et al., 2011).

A large body of evidence has indicated that unbalanced Ca^2+^ homeostasis contributes to the development of neurological and neurodegenerative diseases (Braunewell, 2005; Yu et al., 2009; Demuro et al., 2010; Hermes et al., 2010; Camandola and Mattson, 2011).

Nonetheless, several mechanisms have been developed to control Ca^2+^ homeostasis and to prevent cellular damage. Among these are also nCBPs, which have been reported to display altered levels in neurodegenerative disorders (Steiner et al., 2011). Apart from this role in homeostasis, a major task of many nCBPs is in active signal transduction, where our current knowledge is restricted to only few representatives and is still frequently rudimentary.

Most nCBPs exhibit a tissue specific expression pattern in the mammalian brain. Therefore, they have been used as cell type specific markers before other ways of identification of cell types in the CNS became available (Klausberger et al., 2003). Novel nCBPs are discovered on a regular basis mostly by proteomic screens, each of them with different developmental and cell type specific expression patterns.

A more recently identified CBP is Secretagogin, a multi-faceted protein that is highly expressed in pancreatic beta cells, cells of the gastrointestinal tract as well as in neuroendocrine cells of the CNS (Wagner et al., 2000; Gartner et al., 2001; Mulder et al., 2009). Secretagogin contains six EF-hand motifs as potential Ca^2+^ binding sites, some of which seem to be non-functional. Structural analysis have revealed some similarities with the Ca^2+^ sensor calmodulin (Bitto et al., 2009). Secretagogin undergoes conformational changes upon Ca^2+^ binding indicating a potential role as a Ca^2+^ sensor in specialized cells (Rogstam et al., 2007). Broad screenings by protein arrays have led to the identification of a variety of Secretagogin interaction partners that are associated with vesicle fusion (like SNAP-23, SNAP-25, ARFGAP2, DOC2alpha, rootletin), trafficking (tubulin, KIF5B), enzymatic activity (DDAH-2, ATP-synthase), and one onco-protein (myeloid leukemia factor 2; Rogstam et al., 2007;Bauer et al., 2011a,b). Secretagogin has received considerable attention due to its significance in insulin secretion from pancreatic beta cells and as a potential biomarker for the diagnosis of stroke and distinct tumors of endocrine origin such as adenocarcinomas of the stomach, pancreas, prostate, colorectum, kidney, and lung small cell carcinoma in the blood of patients (Gartner et al., 2001; Lai et al., 2006; Adolf et al., 2007; Ilhan et al., 2011; Zurek and Fedora, 2012). Albeit performed studies on Secretagogin its function is still unclear. By *in silico* gene analysis it was previously found that the Secretagogin gene-promoter sequence might respond to glucose (Skovhus et al., 2006).

Human and mouse brain has been examined to some extent for Secretagogin expression in previous studies (Attems et al., 2008;Mulder et al., 2009, 2010). As the regional expression pattern turned out to differ significantly between species, we now chose to characterize the expression of Secretagogin in rat brain to compare with previous studies on mouse and human brain. In order to perform functional studies we were looking for a primary cell culture system that expresses endogenous Secretagogin at a high level and chose the well-established primary culture system of rat embryonic hippocampal and cortical neurons for *in vitro* studies to get a hint of the expression dynamics and functional aspects of the protein. The tight connection between glucose and insulin, which is also a basic neuronal trophic factor, let us ask, whether insulin and glucose levels influence the expression of Secretagogin in neuronal cells.

Our cell culture system proved to be a suitable model system to perform more functional assays in the future, last but not least, because interference of signaling cascades by drug treatment and tracking the fate and trafficking of proteins by transfection of these neurons with test constructs (like dominant negative or non-functional variants of proteins, fluorescent tagged versions of the protein, and subcellular markers) is technically easy to perform and analysis by common biochemical and cell biological techniques promises relevant results. The *in vitro* studies can be extended to neuronal cultures derived from other brain areas that express high levels of endogenous Secretagogin in order to investigate cell type specific functional similarities or differences of this protein.

Since the recent clinical studies on Secretagogin revealed its potential implication as a novel blood and cerebrospinal fluid biomarker, further knowledge on this protein is of major interest also from the medical point of view.

## Materials and Methods

### Production and purification of secretagogin protein

Human and rat Secretagogin protein was produced and purified the same way as described previously in Wagner et al. (2000) and Gartner et al. (2007), respectively. In brief, the entire coding sequence of the human/rat Secretagogin gene was amplified by PCR using primers with *Eco*RI and *Bam*HI at the flanking end and subsequently cloned into the pGEX-1λT expression vector. The coding sequence for Secretagogin in this construct is located downstream of the coding sequence for glutathione *S*-transferase separated from GST by a thrombin cleavage site. These constructs (GST-SCGN and GST-Scgn) were transformed into *Escherichia coli* BL21, and after colony selection transformed bacteria were grown overnight at 37°C. The next morning the culture was diluted 1:5 and expression of the fusion proteins was induced by addition of isopropyl-β-d-thiogalactoside (final conc. 10 mM) followed by incubation for 3 h at 31°C on a rotating shaker. Afterwards the bacteria were pelleted, sonicated in ice-cold PBS containing 0.1% Triton X-100, and a mix of protease inhibitors. The bacterial lysate was precleared by centrifugation at 12,000 rpm for 10 min and equilibrated glutathione-Sepharose 4B beads were added to the obtained supernatant and mixed for 30 min at 4°C under constant rotation. Following three washes of protein-bound beads with cold PBS, full-length Secretagogin was released by thrombin cleavage. The reaction was incubated for 3 h at RT on a rotating platform, centrifuged at 1500 rpm, and the resulting supernatant containing Secretagogin (human or rat) was tested by SDS-PAGE gel-electrophoresis in order to evaluate the presence of full-length protein. After protein quantification the purified Secretagogin protein was frozen at −80°C until further use.

### Antibodies

Rabbit anti-SCGN antiserum was generated against recombinant Secretagogin protein as described previously and cross-reacts with the rat ortholog (Wagner et al., 2000; Gartner et al., 2001). We used dilutions of 1:1000 for immunohistochemistry and 1:5000 for immunoblotting.

The following commercial antibodies were used for immunofluorescence or other techniques as indicated: rabbit anti-pan-TAU (Cat. No. A 0024, DakoCytomation, Denmark, Glostrup) dil. 1:5000 for immunoblotting; mouse anti-Parvalbumin (Cat. No. PVG214, Swant, Switzerland) dil. 1:3000; mouse anti-Calbindin D28k (Cat. No.300, Swant, Switzerland) dil. 1:10,000; mouse anti-Calretinin (Cat. No. 6B3, Swant, Switzerland) dil. 1:5000; mouse anti-GRP78 (which was a kind gift from the lab of Prof. Johannes Berger) dil. 1:100; mouse anti-GM130 (Cat. No. 560257, BD Biosciences, Franklin Lakes, NJ) dil. 1:100; mouse monoclonal anti-β-actin (AC-15; Cat. No. NB600-501, Novus Biologicals, Littleton, CO, USA) for Western blot controls dil. 1:5000. Secondary antibodies and nuclear staining: DyLight488 goat anti-mouse (Cat. No. 115-485-1460, Jackson ImmunoResearch, Suffolk, UK) dil. 1:400; DyLight488 donkey anti-rabbit (Cat. No. 711-485-1520, Jackson ImmunoResearch) dil. 1:400; Cy3 goat anti-rabbit (Cat. No. 111-166-003, Jackson ImmunoResearch) dil. 1:500; DyLight649 donkey anti-guinea pig (Cat. No. 706-495-148, Jackson ImmunoResearch) dil. 1:400; DyLight649 donkey anti-rabbit (Cat. No. 111-196-003, Jackson ImmunoResearch) dil. 1:400; TO-PRO (Cat. no. T-3605, Invitrogen) dil. 1:500; DAPI (Sigma Cat. No. D8417) final conc. 1 μg/ml in PBS.

### Rabbit anti-SCGN antiserum specificity test

In order to confirm specificity of our rabbit anti-human SCGN antiserum against rat-Scgn, the recombinant purified protein (rat-Scgn, 267 amino acid residues) was pre-incubated with anti-SCGN antibody. The reaction was performed at room temperature for 2 h. The resultant solution containing antibody/antigen complexes was centrifuged at 13,000 rpm for 15 min at 4°C. The supernatant was then used in parallel with native untreated antibody for staining of sections of fixed rat brain. Immunofluorescence staining and imaging was performed as indicated below. The experiment was carried out in duplicates.

Further evidence for antiserum specificity was obtained from a stable cell line expressing human Secretagogin. Jurkat cells were transfected with full-length human Secretagogin encoding plasmid pZeoSV2. Cells, that had stably incorporated the plasmid in the genome and were expressing the protein at a high level were chosen for further experiments.

Protein G-Sepharose beads were used to bind rabbit anti-Scgn antiserum. After washing, beads were incubated with lysates from sham-transfected and SCGN-expressing Jurkat cells. Following three washing steps with 0.1% Tween 20 in PBS (TPBS), beads were eluted in 50 mM triethanolamine, 150 mM NaCl, 0.1% Tween 20, pH 11.2. Eluted fractions were neutralized and loaded onto a 10% SDS-PAGE gel and transferred to nitrocellulose using a semi-dry blotting device. Blotted membranes were blocked and exposed to biotinylated rabbit anti-SCGN antibody followed by incubation with Streptavidine/HRP for 30 min. Each incubation step was followed by two washes with TPBS for 10 min. The blot was finally developed with chemiluminescent reagent and bands were visualized with a Lumi Imager F1.

### Immunohistochemistry of rat brain slices

Six-week-old rats were deeply anesthetized with Equithesin and perfused transcardially with 0.9% NaCl, followed by a mixture of 4% paraformaldehyde, and 15% picric acid in 0.1 M phosphate buffer (PB; pH 7.2–7.4) using a peristaltic pump. Brains were left *in situ* for 10–15 min, removed, and kept in 0.1 M PB with 0.05% sodium azide for a few days at 4°C. Serial coronal sections of 50 μm were cut on the vibratome. Sections were kept in PB containing 0.05% sodium azide at 4°C until staining.

Immunofluorescence experiments were carried out according to previously published procedures (Klausberger et al., 2003; Vasiljevic et al., 2011). Briefly, free-floating sections were incubated in 0.1% Triton X-100/PB for 30 min, blocked in 20% normal horse serum diluted in Tris-buffered saline (50 mM Tris, pH 7.2, 0.85% NaCl) for 2 h, and then incubated in a solution containing a mixture of primary antibodies (rabbit anti-Secretagogin antibody, dil. 1:5000; mouse monoclonal anti-Parvalbumin antibody; mouse monoclonal anti-Calbindin D28k antibody; mouse monoclonal anti-Calretinin antibody) for 48 h at 4°C. Sections were washed and subsequently incubated for 4 h at room temperature with appropriate secondary antibodies conjugated either to Alexa Fluor 488 (anti-rabbit; Invitrogen Molecular Probes, dil. 1:1000) or Cy3 (anti-mouse; Jackson ImmunoResearch Laboratories, dil. 1:400). All antibodies were diluted in TBS containing 0.1% Triton X-100 and 1% normal horse serum. After washing in TBS (50 mM Tris buffer, pH 7.4), sections were mounted in Aqua PolyMount (Polysciences Europe, Eppelheim, Germany), and left to polymerize at 4°C over night. Sections were examined with a Leica TCS SP5 II confocal microscope (Leica Microsystems GmbH, Wetzlar, Germany). All antibodies were tested for optimal dilution, and secondary antibodies were tested for cross-reactivity and non-specific staining.

### Immunohistochemistry of human samples

Brain tissue obtained from routine autopsy was fixed in 4% Formalin and embedded in paraffin as a routine procedure for the process of pathological specimens. Tissue sections of 4 mm were deparaffinized with xylol, passed through a graded ethanol series and finally washed in distilled water. Endogenous peroxidase was blocked with 3% H_2_O_2_ in methanol for 10 min. Following antigen retrieval in citrate buffer, pH 6.0 for 60 min at 96°C, slides were washed in Tris-buffered saline (TBS), and blocked with 10% FCS for 10 min. The primary antibody was applied for 2 h. After washing the slide for 10 min in PBS the secondary antibody was applied and incubated for 1.5 h at RT. Following two washing steps for 10 min at RT in PBS, the chromogenic substrate DAB was applied (EnVision ^™^Kit DakoCytomation K5007) and the reaction was stopped after 10 min by washing the slide with tap water. As a counterstain Mayer’s Hemalaun was used for 30 s, which was washed off by tap water. Finally, slides were exposed to 0.45 M HCl in 70% EtOH followed by a further wash in water. Sections were dehydrated in acetyl butyrate and mounted with a coverslip. Images were taken using a Leica Aristoplan microscope.

### Primary rat cell culture

Primary neuronal cultures were prepared from rat E18 embryos (pregnant Sprague-Dawley rats were purchased from Charles River, Germany). Procedures were carried out in accordance with animal care guidelines of the Medical University of Vienna, Austria. Cultures were essentially prepared as neuron/glia co-cultures as described previously with modifications (Brewer et al., 1993). Hippocampus and cortex were dissected and individually trypsinized at 37°C in a 10 mM HEPES buffered Hank’s balanced salt solution (HBSS; GIBCO Life Technologies, NY, USA). Tissues were washed three times with ice-cold HBSS and dissociated by trituration. Cortical neurons were additionally sieved through 100 and 70 μm filters (Falcon). Neurons were plated at a density of ∼52,500 cells/cm^2^ into 24-well culture plates. For Western blot analysis and real-time quantitative PCR (RT-qPCR) six-well culture plates were seeded at the same density. Cells were left to attach overnight in MEM medium containing 10% horse serum, 1 mM sodium pyruvate (all from GIBCO), 1.2% d-glucose (Sigma), 100 U/ml penicillin together with 100 μg/ml streptomycin (GIBCO). All surfaces were coated with a solution of 0.5 mg/ml poly-l-lysine (MW 30,000–70,000; Sigma) in 0.05 M sodium borate, pH 8.0 overnight at room temperature and were washed thoroughly with distilled water before adding the culture medium. Cultures were maintained in an incubator with 5% CO_2_ at 37°C during the whole culturing period. After attachment of cells, the medium was replaced by serum-free Neurobasal medium (GIBCO) supplemented with 2% v/v B-27 (Invitrogen), 2 mM glutamine (GIBCO), 1.2% d-glucose (Sigma), 100 U/ml penicillin, and 100 μg/ml streptomycin.

Hippocampal neurons were used for experiments mostly between 14 and 22 DIV (days *in vitro*), whereas cortical neurons were used at 7 DIV.

### Cell culture conditions for studies with insulin

Hippocampal neurons were grown in six-well culture plates either in normal Neurobasal medium supplemented with 2%v/v B-27, 2 mM glutamine, 1.2% d-glucose, 100 U/ml penicillin and 100 μg/ml streptomycin, or in insulin-free medium consisting of Neurobasal medium and all ingredients such as indicated above, except commercially available insulin-free B-27 (Cat. No. 05-0129SA, GIBCO). For insulin deprivation experiments neurons were initially grown in standard Neurobasal medium in order to support optimal growth. At 18 DIV, one third of medium was replaced with pre-warmed insulin-free medium. This procedure was repeated on the following 2 days. On the fourth day neurons were harvested using TRIzol reagent (Cat. No. T9424, Sigma, Germany) to stabilize the mRNA and samples were frozen at −80°C until further proceeding. For insulin boost experiments 21 DIV neurons grown in standard Neurobasal medium were challenged once with 100 μg/ml of insulin (Cat. No. I9278, Sigma, Germany) added to the growth medium for 1, 4, and 24 h. After the individual time periods, neurons were again harvested with TRIzol reagent. Additionally, we cultured hippocampal neurons in insulin-free medium from 0 DIV onward in comparison with the same batch of cells in standard growth medium and harvested the cells at 21 DIV using TRIzol reagent.

### Isolation of mRNA from rodent and human brain samples

Three rats were sacrificed and their brains were isolated. Tissues were immediately shock frozen in liquid nitrogen. Subsequently, different brain regions (cerebellum, hippocampus, striatum, frontal cortex, parietal cortex, olfactory bulb) were dissected under the microscope.

For human samples tissues were obtained from the Pathology Department, Medical University of Vienna, Austria in agreement with the Ethical Committee of the Medical University of Vienna (EK: 987/2010). Samples were from three male individuals 62, 73, and 70 years of age, who have died of lung cancer, chronic obstructive airway disease and myocardial infarction, respectively.

About 30 mg of each human brain tissue (cerebellum, frontal cortex, parietal cortex, occipital lobe, temporal cortex, hippocampus, olfactory bulb, thalamus, hypothalamus, stem ganglia) was put into TRIzol reagent and RNA was isolated as described below.

### Real-time quantitative PCR

RNA (from neuronal cells or brain tissues stabilized with TRIzol reagent) was extracted using chloroform-isopropanol with phase-separation by centrifugation. Subsequently, 1 μg of total RNA was reverse-transcribed (1 h, 55°C) using MLV reverse transcriptase (Invitrogen). The diluted (1:3) cDNA was used as a template together with TaqMan 2xMasterMix (Lot: N10545, Applied Biosystems) and with TaqMan probes specific for rat-Scgn (No. Rn01529973_m1), human-SCGN (No. Hs00907373_m1), rat-Tau (No: Rn01495715_m1), rat-SNAP-25 (No. Rn00578534_m1), rat-Syt-1 (No. Rn00436852_m1), rat-Sgk (N0. Rn00570285_m1), and rat-Ins-1 (No: Rn02121433_g1). The RT-qPCR measurement was carried out at the StepOnePlus Fast Real-Time PCR System (Applied Biosystems). Expression values were calculated according to the ΔΔCT method or with calculation of the copy number using rat-Scgn plasmid as standard. As housekeeping genes, the TaqMan Ubc probe (No. Rn01789812_g1) was used for samples from neuronal cell cultures and the Gapdh probe (No: Rn01775763_g1 for rat, Hs02758991_g1 for human) was used in brain samples.

### GST pull-down assay from rat brain

GST pull-down assays were performed essentially in the same manner as described in (Maj et al., 2010). Briefly, human GST-SCGN fusion protein or GST only was loaded onto Glutathione-Sepharose 4B beads (Cat. No.17–0756–01, Healthcare Biosciences). Whole rat brain tissue was homogenized and 200 mg was lysed in 2000 μl TPBS including 1 mM PMSF in Lysing Matrix A (MP Biomedicals Cat.nr. 6910-050) tubes using the Precellys 24 lysis and homogenization device set at 5000 for 20 s. The resultant lysate was centrifuged at 12,000 × *g* for 10 min and incubated with GST- and GST-SCGN-Sepharose beads according to Maj et al. (2010). In brief, 35 μl of beads were incubated with 400 μl of lysate at 4°C for 90 min under constant rotation. In order to test for calcium dependence of the interaction between Secretagogin and Tau protein, we used different concentrations of EDTA (0, 2.5, 5.0, 7.5, and 10.0 mM) in the lysate upon incubation with GST-SCGN-Sepharose beads. After three washes with TPBS including 1 mM PMSF bound proteins were eluted with 10 mM EDTA. The eluates were then further processed for Western blot analysis. 15% of the lysate used for pull-downs was loaded as input control.

### Immunoprecipitation

PureProteom protein G magnetic beads (100 μl, Millipore) were washed in PBS and loaded with D24 mAb by incubation at RT for 60 min under constant rotation (negative control: unrelated isotype mAb) and afterwards washed with 0.2 M sodium borate pH 9.0. Antibodies bound to the beads were chemically crosslinked using 40 mM dimethyl pimelidate dihydrochloride (DMP, Sigma) for 1 h at RT. Excess of DMP was neutralized with 0.2 M ethanolamine pH 8.0 followed by washes with PBS. Before use, beads were pre-eluted with 0.1 M glycine pH 2.0.

For immunoprecipitation, antibody-coupled beads were incubated with brain extract (prepared as for GST pull-down experiments) and incubated at 4°C for 2 h under constant rotation. After three washes with TPBS, bound protein was eluted with 100 mM glycine pH 2.0. Eluates were loaded onto SDS-PAGE, transferred onto nitrocellulose and developed with rabbit anti-SCGN and anti-pan-Tau, respectively.

### Subcellular fractionation on a sucrose gradient

One-hundred milligrams of whole rat tissue were homogenized in 1.7 ml sucrose buffer (250 mM sucrose, 20 mM Tris/HCl pH 7.4, 1 mM EDTA, protease inhibitors). Cell nuclei and large aggregates were pelleted by centrifugation at 10,000 × *g* for 10 min. The entire supernatant was loaded onto a discontinuous sucrose gradient in polyAllomer centrifuge tubes (14 mm × 95 mm, Beckman). Following centrifugation (2 h at 4°C) at 40,000 rpm using a SW40 Ti rotor in a L-80 ultracentrifuge, the gradient was fractionated in 500 μl aliquots using a peristaltic pump starting at the bottom of the tube. Individual fractions were subjected to immunoblotting using antibodies for Scgn, SNAP-25, α-tubulin, and pan-Tau.

## Results

### Expression of secretagogin in the mammalian brain

**Figure 1:**
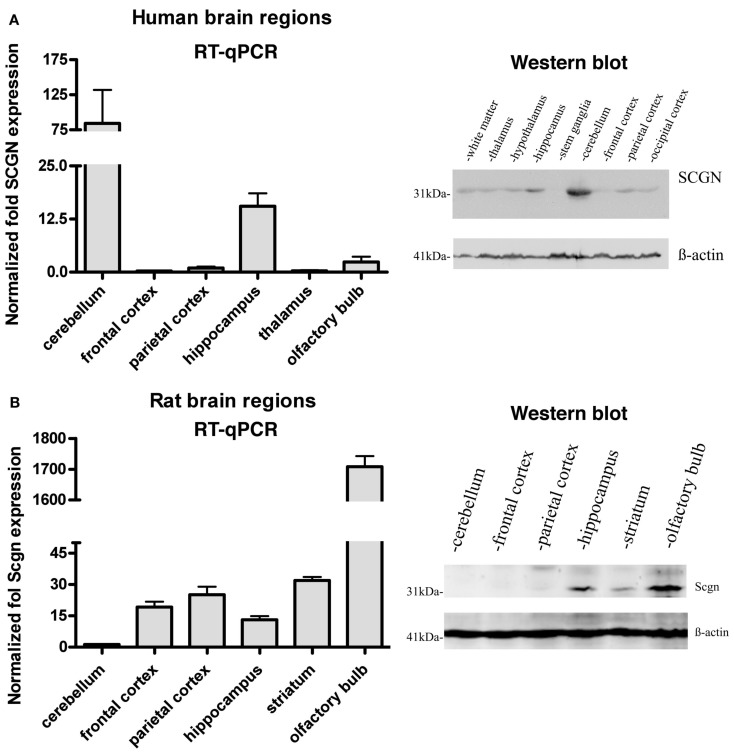
**Expression of Secretagogin in different regions of human and rat brain**. Tissues from different brain areas were analyzed for relative Secretagogin gene and protein expression by RT-qPCR and Western blotting. **(A)** Human post-mortem tissues from three individuals were analyzed with regard to the following specific brain regions: cerebellum, frontal cortex, parietal cortex, hippocampus, thalamus, and olfactory bulb. Western blot analysis was performed from tissue of white matter, thalamus, hypothalamus, hippocampus, stem ganglia, cerebellum, frontal cortex, parietal cortex, and occipital cortex. Equal protein loading was verified by staining with β-actin antibody. **(B)** Rat brain tissues from three adult rats were dissected and analyzed from cerebellum, frontal cortex, parietal cortex, hippocampus, striatum, and olfactory bulb. A representative Western blot of equal amounts of protein from cerebellum, frontal cortex, parietal cortex, hippocampus, striatum, and olfactory bulb is shown on the right. β-actin immunostaining was again used as loading control.

**Figure 2:**
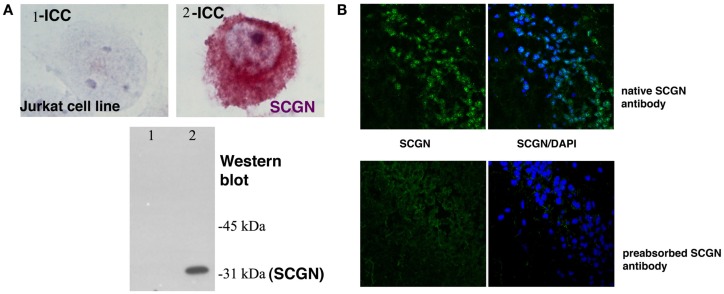
**Secretagogin antibody specificity**. **(A)** A Jurkat cell line (1) was stably transfected with human Secretagogin (2). Immunocytochemistry and Western blot analysis from cell lysates reveal the specificity of the antibody in this heterologous cell line for both techniques. **(B)** Secretagogin antibody specificity test on rat brain slices: our Secretagogin antibody was used to stain rat brain slices in the CA1 region of the hippocampus without (upper panel) and with pre-absorption (lower panel) with purified antigen. Secretagogin staining is shown in green and nuclear staining (DAPI) in blue.

Expression of Secretagogin has been found at the highest level in pancreatic beta cells and in neuroendocrine cells of the CNS (Wagner et al., 2000; Gartner et al., 2001; Attems and Jellinger, 2006; Mulder et al., 2010). Studies on mouse and human brain tissue indicated a varying expression pattern in mice and men. However, until now no detailed comparative analysis of the Secretagogin distribution in mammalian brain has been presented. We used quantitative real-time PCR (RT-qPCR) to measure relative Secretagogin mRNA levels in different major areas of rat brain and compared it to tissue from the corresponding areas in human brain (Figure 1). Interestingly, while human brain reveals an expression maximum in the cerebellum (Figure 1A), in rat brain by far the highest expression of Secretagogin is found in the olfactory bulb (Figure 1B). This is also the case in mouse brain (Mulder et al., 2009). Significant expression of Secretagogin is also found in the hippocampus of humans and rodents confirming previous studies (Gartner et al., 2001; Attems et al., 2007; Mulder et al., 2009). The same pattern translates into differences of protein expression especially with regard to expression maxima in cerebellum and olfactory bulb respectively, which we confirmed by Western blotting also for human and rat brain (Inserts in Figures 1A,B). Differences between mRNA levels and protein have been found in other areas with lower expression levels. β-actin was used as internal standard. For all of our immunological studies we used a rabbit polyclonal anti-SCGN antibody generated in our lab. The antibody specificity has been tested for individual applications, like Western blotting, immunocytochemistry of fixed cells in culture, and immunohistochemistry on paraffin-embedded human and rat tissue and paraformaldehyde-fixed rat tissue (Figures 2A,B).

### Cell type specific expression of secretagogin in human cerebellum and rat olfactory bulb

**Figure 3:**
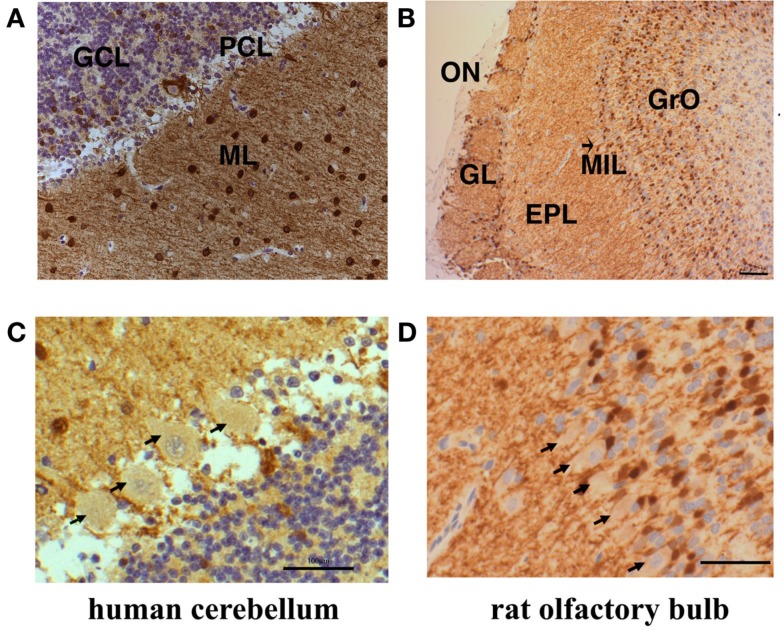
**Subcellular distribution of Secretagogin-positive cells in human cerebellum and rat olfactory bulb**. Paraffin sections from human cerebellum **(A)** and rat olfactory bulb **(B)** were processed for Secretagogin immunoreactivity (DAB staining in brown) and counterstained with Mayer’s Hemalaun (blue). Human cerebellum reveals positive Secretagogin staining of interneurons in the molecular layer (ML) and the granule cell layer (GCL). Granule cells are immuno-negative and Purkinje cells bodies revealed faint positive staining (PCL). Actual expression of Secretagogin in Purkinje cells would need verification by single cell analysis. The rat olfactory bulb shows Secretagogin-positive cells in the glomerular layer (GL), the external plexiform layer (EPL), and the granular cell layer (GrO). The mitral cell bodies (indicated by an arrow, MIL) seem to be very weakly positive. Scale bars: 200 μm. Magnifications of the human Purkinje cell layer and rat mitral cells are shown in **(C,D)** respectively. Principal cells are indicated by arrows. Scale bars: 20 μm.

The neuronal circuits and cell identities are well defined in cerebellum and olfactory bulb. In order to investigate the distribution of Secretagogin in the different cell types of human cerebellum and rat olfactory bulb we performed immunohistochemistry from paraffin-embedded slices of the respective tissues. Human cerebellum reveals especially high Secretagogin expression in the molecular layer (Figure 3A). Positive neurons have previously been identified as interneurons of the basket and stellate cell type (Gartner et al., 2001). Purkinje cell bodies are faintly staining positive, granule cells are free from intracellular Secretagogin staining, while interneurons of the granule cell layer are Secretagogin-positive (Figures 3A,C). A contribution of a small cross-reactivity of our Secretagogin antibody to faint stainings (like in Purkinje cell bodies) cannot be fully excluded.

The outer part of the olfactory bulb is also organized in well defined cell layers. In rat, many neurites (probably dendrites and axons) and some cell bodies stain positive in the glomerular layer and in the external plexiform layer. However, within the granular cell layer mainly cell bodies and only few neurites are Secretagogin-positive. From the routine experience with our antibody we consider the faint staining of mitral cell bodies as slightly positive for Secretagogin. But a staining due to weak cross-reactivity again cannot be fully excluded (Figures 3B,D).

### Hotspots of secretagogin-positive cells in specialized areas of rat brain

As previous studies describe Secretagogin in the mouse and human nervous system, we extended our investigations to rat brain. Although both mice and rats are rodents, differences in Secretagogin distribution cannot be excluded. Also, recent studies on mouse focused only on special areas in the mouse brain (Mulder et al., 2009, 2010).

**Figure 4:**
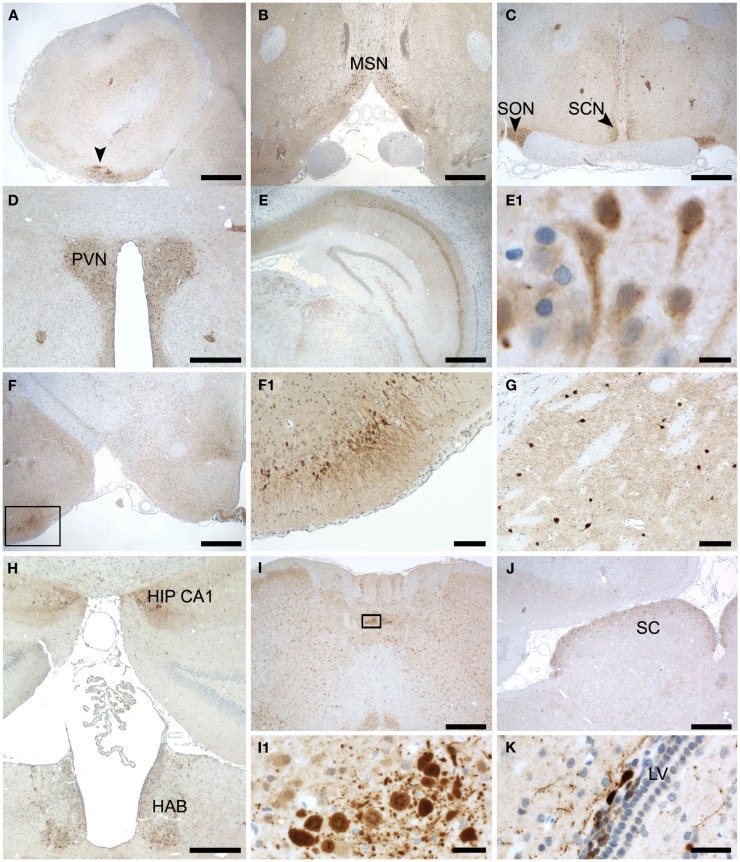
**Subregional expression of Secretagogin in rat brain**. Representative sections from rat brain were stained for Secretagogin immunoreactivity and counterstained with hematoxylin. Panels represent the following brain areas: **(A)** olfactory bulb, arrow pointing to a patch of strong Scgn+ cells. Scale bar: 500 μm. **(B)** Medial septal nucleus. Scale bar: 500 μm. **(C)** Supraoptic nucleus (SON) and Suprachiasmatic nucleus (SCN). Scale bar: 500 μm. **(D)** Paraventricular nucleus (PVN). Scale bar: 400 μm. **(E)** Hippocampus. Scale bar: 500 μm. **(E1)** Magnification of hippocampal neurons in CA1. Scale bar: 10 μm. **(F)** Lateral amygdaloid nucleus (indicated by box). Scale bar: 500 μm. **(F1)** Magnification of the region indicated in **(F)**. Scale bar: 100 μm. **(G)** Cells in caudate putamen. Scale bar: 100 μm. **(H)** Panel reveals two Scgn+ areas: beginning of hippocampal CA1 region (HIP CA1) and habenula (HAB). Scale bar: 400 μm. **(I)** Medulla oblongata. Scale bar: 500 μm. **(I1)** Magnification of a strongly Scgn+ nucleus in medulla oblongata as indicated in **(I)**. Scale bar: 25 μm. **(J)** Superior colliculus (SC). Scale bar: 500 μm. **(K)** Cells around the lateral ventricle. Scale bar: 25 μm.

**Figure 5:**
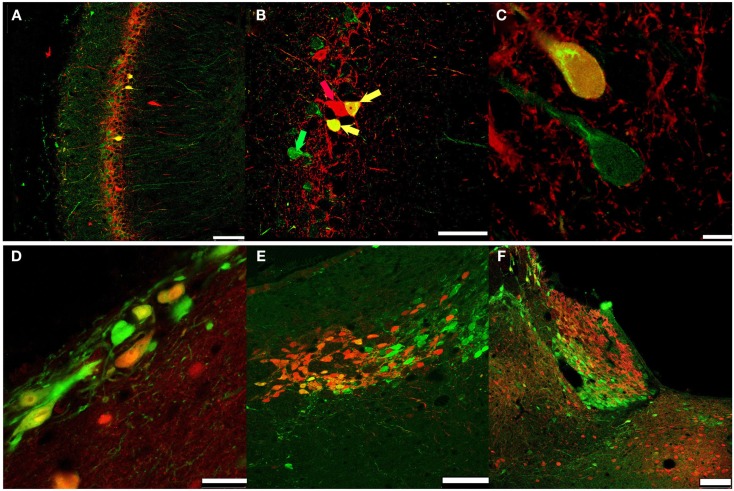
**Secretagogin and other CBPs**. PFA-fixed rat brain slices were immunostained for Secretagogin (Scgn: green) and other CBPs (red) and processed for immunofluorescence. **(A-C)** Hippocampal CA1 region: Secretagogin (green), Parvalbumin (PV, red). **(A)** Scale bar: 100 μm**. (B)** Arrows indicate co-expression of Scgn and PV; green arrow: Scgn only, red arrow: PV only; yellow arrow: co-expression of Scgn and PV in a single neuron. Scale bar: 50 μm. **(C)** Larger magnification of Scgn/PV co-expressing neurons indicates a distinct subcellular distribution. Scale bar: 7.5 μm. **(D)** Secretagogin-positive neurons near the ventricle. Secretagogin (green), Calbindin D28k (red). Scale bar: 25 μm. **(E)** Habenula: Secretagogin (green), Calbindin D28k (red). Scale bar: 75 μm. **(F)** Habenula: Secretagogin (green), Calretinin (red). Scale bar: 100 μm.

Therefore we stained coronal sections from rat brain with anti-Secretagogin antibody and counterstained with hematoxylin (Figure 4). Secretagogin-positive (Scgn+) cells frequently occur in cell clusters in addition to positive cells scattered over larger areas. As described for mouse, also in rats peripheral cell layers of the olfactory bulb are Scgn+. When choosing a more lateral layers in addition to the cell layers described in Figure 3, seemingly unordered patches of strong Scgn+ cells were observed (Figure 4A). Moving in caudal direction we found strong staining lining the surface above the optic nerve (Figure 4B). Further positive nuclei are the supraoptic nuclei on both sides of the optic chiasm and the suprachiasmatic nuclei of the hypothalamus (Figure 4C). In addition some islets of clustered cells can be seen in this figure, which we could not unambiguously attribute to a defined anatomical structure (arrows). A highlighted area is also the paraventricular nucleus (Figure 4D). Prominent Secretagogin staining has been observed in hippocampal areas CA1–CA3, with almost no staining in dentate gyrus (Figure 4E). A magnification of an area in CA1 reveals staining of hippocampal pyramidal cell bodies and more positive intercalated interneurons as defined later in co-stainings with antibodies for marker proteins in Figure 5 (Figure 4E1). Moving further in caudal direction again accumulations of strongly stained cells were detected in the outer cortex around the posterolateral cortical amygdaloid area (Figure 4F). A magnification of the positive cell patch reveals strong staining of neuronal processes in this area (Figure 4F1). Further a meshwork of positive processes with some scattered Scgn+ cells is observed in the basal ganglia with caudate putamen shown in (Figure 4G). Patches of positive cells are seen at the beginning of the hippocampal formation close to the ventricle. The same figure reveals Scgn+ cells in the habenular region, which is located ventral to the hippocampus, again near the ventricle (Figure 4H). The lateral amygdala nucleus reveals also quite a few positive cells (not shown). Two nuclei, arrays of surface lining cells and scattered Scgn+ cells were found in the medulla oblongata (Figure 4I and magnification in Figure 4I1). Finally we also found the superior colliculus as a Scgn+ area (Figure 4J). Larger magnifications of Scgn+ cells in different areas are shown in (Figures 4K,E1).

### Secretagogin and other calcium binding proteins

Coronal sections revealing the hippocampal formation were co-stained for Secretagogin and other known CBPs like Parvalbumin, Calbindin D28k, and Calretinin (Figure 5).

The antibodies mostly recognized different cell populations with occasional overlaps like co-expression of Parvalbumin and Secretagogin in interneurons of the CA1-3 regions (Figures 5A–C), and Calbindin-Secretagogin co-expression in young, possibly migrating cells (Figure 5D). Frequently, patches of Secretagogin-positive cells are in close vicinity of patches of neurons that stain positive for another CPB. This can nicely be shown in the habenula, where large groups of Secretagogin-positive cells are flanked by groups of Calbindin or Calretinin-positive cells (Figures 5E,F).

### Subcellular localization of Secretagogin

We observed some degree of colocalization of Parvalbumin-positive (inter-)neurons with Secretagogin in rat hippocampus. But cells, that co-expressed Parvalbumin and Secretagogin mostly revealed a different subcellular staining pattern for both proteins (Figures 5A–C). While Parvalbumin always stained the soma homogenously, Secretagogin frequently seemed to be accumulated on subcellular structures (Figure 5C). In order to visualize the different subcellular distribution we performed a high resolution z-stack with a confocal microscope of a Parvalbumin/Secretagogin-positive cell from a rat brain slice in the CA1 region of the hippocampus and show the three-dimensional reconstruction in a movie (see Movie S1 in Supplementary Material).

**Figure 6:**
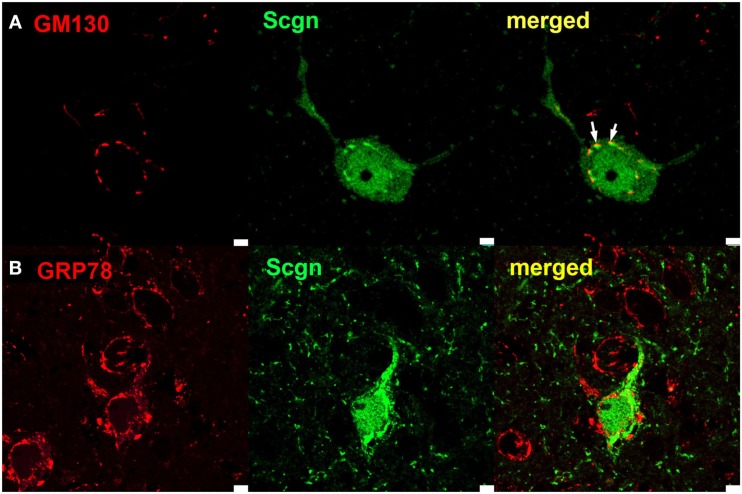
**Subcellular localization of Secretagogin**. Rat brain slices were processed for immunofluorescence with anti-Secretagogin antibody (green) and antibodies for marker proteins of subcellular compartments (red): **(A)** GM130 (for cis-Golgi compartments) and **(B)** GRP78 (for Endoplasmatic Reticulum). White arrows in upper panel indicate close spatial apposition of Secretagogin clusters and GM130. Scale bar: 10 μm.

To further investigate the structures, that Secretagogin seems to be associated with, we stained for compartmental maker proteins localized in the ER or Golgi apparatus. The marker GM130 is an intracellular peripheral membrane protein associated with the cis-Golgi network and vesicles derived from ER and associated with cis-Golgi. We found, that Secretagogin frequently stained clusters in immediate apposition to GM130 clusters. In contrast, Secretagogin clusters seemed not to be spatially associated with staining for the ER marker GRP78 (Figure 6). The close localization of Secretagogin and GM130 is again visualized in a three-dimensional reconstruction based on z-stack imaging (see Movie S2 in Supplementary Material).

**Figure 7:**
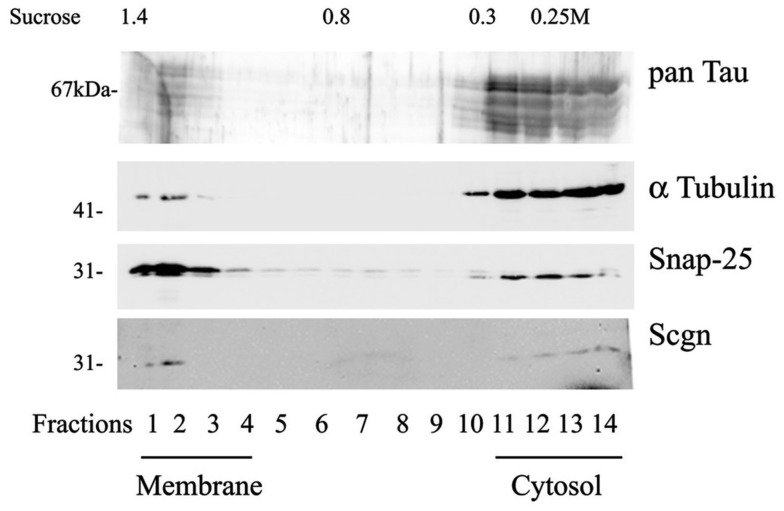
**Fractionation of rat hippocampal extract by sucrose density gradient**. The fractionation and sampling was done as described in the methods section. Fractions were immunoblotted and proteins detected with antibodies for Tau protein (pan-Tau), alpha-tubulin, synaptic vesicle-associated protein SNAP-25 and Secretagogin. Fractions of high density hold the membrane-associated components and fractions with lower sucrose concentration contain soluble proteins of the cytosol. Calculated sucrose concentrations of collected aliquots are indicated on top of the panel. The experiment was repeated three times with similar results.

We also used sucrose density centrifugation of rat hippocampal tissue extracts to separate membrane-bound proteins from soluble proteins. Secretagogin was found in the membrane-fraction as well as in the cytosolic fraction (Figure 7).

### Secretagogin and Tau protein

**Figure 8:**
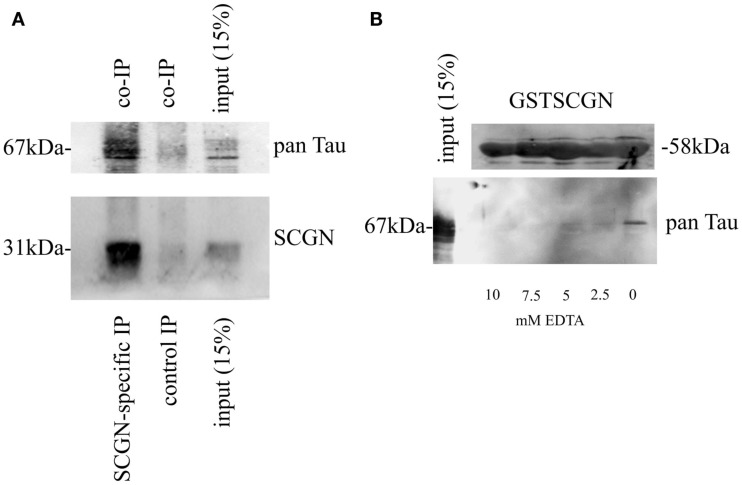
**Calcium-dependent association of Secretagogin with Tau protein**. **(A)** Immunoprecipitation from brain extracts using anti-Secretagogin antibody followed by immunoblot with pan-Tau antibody (upper panel) and anti-Secretagogin antibody (lower panel). The input-lane contains 15% of the whole extract used for immunoprecipitation. For a negative control an equal amount of an unrelated antibody was used for the immunoprecipitation. **(B)** Pull-down experiment from rat brain using GST-SCGN fusion protein in the presence or absence of EDTA. Upper panel: Coomassie staining of GST-SCGN fusion protein, which was used for pull-down assays. Lower panel: Immunoblot with pan-Tau antibody. The input-lane contains 15% of the extract used for individual pull-down experiments. All experiments were repeated at least three times.

Secretagogin has previously been shown to interact with an isoform of Tau in an insulinoma cell type (Maj et al., 2010). We now investigated, if this interaction is also present in rat brain. We immunoprecipitated Secretagogin from brain extracts and performed a Western blot. The co-precipitation of Tau was confirmed by incubation with a pan-Tau antibody (Figure 8A).

The dependence of this interaction on the presence of Ca^2+^ was verified with an *in vitro* pull-down assay. A GST-fusion protein of full-length Secretagogin was incubated with rat brain extracts in the absence or presence of different concentrations of EDTA. Tau protein could only be found to be associated with GST-SCGN in the complete absence of EDTA, indicating the Ca^2+^-dependence of this interaction (Figure 8B).

### Insulin regulates Secretagogin expression *in vitro*

Insulin is a conventional growth factor for cultured cells. A vast number of publications deals with the roles of insulin in the nervous system affecting neuronal growth, neuroprotection, neurotransmitter release, activation of Ca^2+^ channels, and more (Unger et al., 1991; Zhao and Alkon, 2001). On the other hand, Secretagogin has been discovered and is now well-established as Ca^2+^ sensor in insulin secretion from pancreatic beta cells (Wagner et al., 2000). Interestingly, Secretagogin mRNA transcript levels are significantly higher in a pancreatic tissue obtained from Goto–Kakizaki rats (an animal model for type 2 diabetes) when compared to non-diabetic control Wistar rats (Bazwinsky-Wutschke et al., 2010). On this basis, we aimed to investigate the effects of insulin on the expression of Secretagogin in a rat primary neuronal cell culture.

Hippocampal neurons were grown in standard Neurobasal medium with B-27 supplement (with a basal insulin concentration of 40 pg/ml in the final medium as indicated in the data sheet of the manufacturer). When synaptogenesis was finished, a strong insulin boost (final concentration: 100 μg/ml) or a gentle – but almost complete – insulin deprivation was performed as described in the Section “Materials and Methods.”

**Figure 9:**
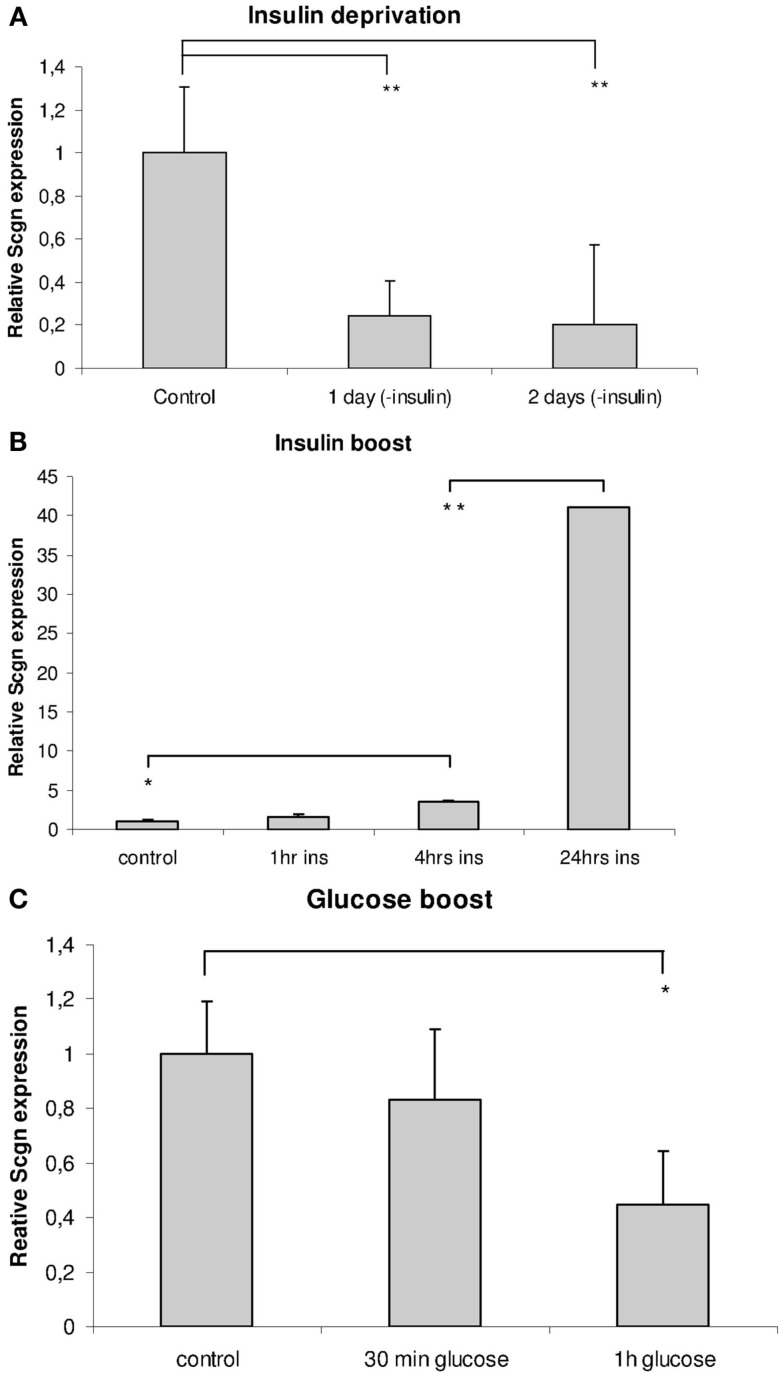
**Insulin and glucose regulate Secretagogin expression in neuronal cell culture**. Hippocampal neurons in culture were subjected to insulin deprivation or challenged with an insulin- or glucose-boost and analyzed for Secretagogin expression at 21 DIV. Relative amounts of Secretagogin mRNA were determined by RT-qPCR after insulin deprivation **(A)**, insulin boost **(B)**, and glucose boost **(C)** at different time points after treatment (*n* = 3).

Insulin deprivation from normal culture medium over 3 days caused a significant decline in Secretagogin gene expression level to as little as 20% of control levels already on the first day after feeding with insulin-free Neurobasal/B-27 medium (Figure 9A), whereas a single insulin boost caused a steady highly significant increase in its expression levels after 24 h (Figure 9B). In addition, we measured the mRNA level of other proteins similarly involved in exocytosis processes, such as SNAP-25 and synaptotagmin-1. These levels were also significantly increased by the insulin boost (not shown). All measurements were related to housekeeping genes.

As insulin is a major control factor of the energy status, we also checked if addition of a high glucose concentration to the medium has an effect on Secretagogin gene expression level. We expected that the effect of glucose would be rather fast, as it is readily taken up by cells via transporters and metabolized quite fast. One hour after the addition of glucose to a final concentration of 50 mM we harvested the cells and determined the expression level of endogenous Secretagogin mRNA, which had significantly declined by 56%. The data from the glucose-boost experiments show that glucose decreases Secretagogin expression (Figure 9C).

## Discussion

In the present study we report novel immunocytochemistry and functional data of the CBP Secretagogin in the context of neuronal cells. Secretagogin has been shown to be involved in insulin release from pancreatic beta cells, but it is also highly expressed in neurons – another type of excitable cells. Studies in flies have revealed that the pancreatic islet has evolutionarily developed from an ancestral insulin-producing neuron (Rulifson et al., 2002; Craft and Watson, 2004). The intrinsic properties of the protein might be similar in different locations, but the functional aspects most probably have evolved independently in the endocrine and nervous system. A large number of CBPs exist with different molecular properties. Some CBPs have been used as markers for neuronal cell types especially to group the enormous heterogeneity of cells in the nervous system before other measurable characteristics like unambiguous morphologies or firing patterns became accessible. More recently, those CBPs received focused attention with regard to their individual functional properties. Their very limited co-localization indicates, that different cell types might tune the expression according to their needs based on the molecular properties of the CBPs.

Secretagogin belongs to a family of hexa EF-hand CBPs and is capable of binding four Ca^2+^ ions at physiological intracellular Ca^2+^ levels with a binding affinity of Ca^2+^ similar to other nCBPs (Rogstam et al., 2007). High Secretagogin expression was reported not only in the pancreatic tissue, but also in subpopulations of developing or adult neurons (Gartner et al., 2001; Mulder et al., 2009, 2010). As the expression of Secretagogin is restricted to specialized brain areas, it is especially motivating to research for a context of Secretagogin expression and functional aspects of these neuronal populations in the organism. Intrinsic mechanisms of activation (also with regard to Mg^2+^ as a co-factor), subcellular location, and target protein specificity as investigated also for other CBPs are important parameters in this regard (Rogstam et al., 2007; Schmidt, 2012).

In this study we characterized the distribution of Secretagogin immunoreactivity in rat brain and in specialized areas from human brain. The unique, but distinct Secretagogin expression pattern within human and rodent brain presumably underlines the existence of complex and refined mechanisms of Ca^2+^ signaling. This goes hand in hand with the expression of neuronal insulin and insulin receptors. Apart from entering the CNS via the blood-brain barrier by a receptor-mediated transport process, insulin expression is also found directly from cells of the CNS. In rodents insulin binding is especially high in the olfactory bulb, the cerebral cortex, hippocampus, hypothalamus, amygdala, and septum (Baskin et al., 1987). Insulin receptors are also found in the substantia nigra, basal ganglia, and frontal cortex (Unger et al., 1991; Craft and Watson, 2004). By immunohistochemistry we could show a high expression of Secretagogin not only in the olfactory bulb and the hippocampal pyramidal cell layer, but also in the basal ganglia, and with extreme density in major nuclei of the hypothalamus (in the neurosecretory neurons of the supraoptical nucleus, the suprachiasmatic nucleus controlling circadian rhythms, and the paraventricular nucleus involved in the control of food-intake), the habenula, the amygdala, the medulla oblongata, and the superior colliculus. Many neurons in the hypothalamus release peptide hormones like oxytocin, vasopressin, vasoactive intestinal peptide, anti-diuretic hormone, and others that are released into the blood as well or act directly within the brain on receptors on the surface of other neurons. Many of these areas have some functional similarities and are interconnected. In analogy to insulin-releasing pancreatic beta cells, a role of Secretagogin in neuronal hormone-release or glucose metabolism can be hypothetically anticipated. The habenula is a phylogenetically old area receiving input from the limbic system, the pineal gland, and the basal ganglia. The habenula is thought to play a part in many basic processes. It is a relay area for olfactory stimuli and is thought to be involved in processing of external stress, decision-making, and also sleep (Hikosaka, 2010). The habenula seems to be especially activated during experiences that are associated with unpleasant events, the absence or opposite of reward, and even punishment. In rat models of depression the glucose metabolism in the lateral habenula has been shown to be elevated. Secretagogin knock-out mice would be an extremely interesting model system for investigating the role of this CPB in more complex brain-body functions.

In relation to other CBPs, we observed only a restricted overlap of Secretagogin with Parvalbumin and Calbindin D28k in some areas. Even in cells expressing Secretagogin together with another CBP, the time course of expression or the subcellular localization might be different in development, different phases of brain activity or metabolic states. A role of Secretagogin in developing or not terminally differentiated neurons has been proposed and shown previously in some areas like the olfactory bulb (Mulder et al., 2009, 2010). In our studies we also observed Secretagogin-positive neurons, that are probably not terminally differentiated in areas known to contain migrating neurons (as shown in Figure 4K) and sometimes also in areas with few other neuronal cell bodies like the corpus callosum (not shown). We think, that this issue needs a detailed study on its own. It might be possible, that neurons also switch the expression of their CBPs in the course of development.

In mature neurons of the CA1 pyramidal cell layer a compartmental association of Secretagogin could be observed in rat brain slices. Secretagogin does neither possess a classical signal peptidase cleavage site that would be a sign for targeting to the ER nor a consensus site for myristoylation for membrane attachment like in many other CBPs. However, we could show a direct opposition of strong Secretagogin and GM130 immunoreactivity indicating an association with cis-Golgi vesicles or compartments. The exact identification of these compartments and the form of interaction needs further investigations.

It is accepted knowledge that Ca^2+^ homeostasis is shifted during normal aging and is especially disturbed in many neurodegenerative disorders. Thus the intimate connection between intracellular Ca^2+^ levels and diseases of the CNS reinforces the importance of maintaining the delicate balance of Ca^2+^ levels within the brain, and the important role of CBPs in this matter. Of special interest with regard to Alzheimer’s disease further studies on the Secretagogin-Tau interaction *in vitro* should not be neglected. A neuroprotective role of Secretagogin in the human hippocampus has been previously suggested, as Secretagogin expressing neurons have been shown to be free of the pathological hyperphosphorylated form of Tau in AD brains (Attems et al., 2008). In particular the Secretagogin expression level in human hippocampus from AD patients versus healthy individuals seems to remain unchanged for longer periods of the developing illness, implying that these neurons might be more resistant to pathological deregulation and cell death (Attems et al., 2008). A causal link between Secretagogin expression and neuroprotection has not been shown. But also, if this hypothesis holds true, a contribution of other factors might as well play a significant role. Unpublished observations indicated that Secretagogin seems to be upregulated in cells close to amyloid plaques what can be interpreted as a protective adaptation with the aim of regeneration. This promising hints have led to further studies on the communication between Secretagogin and Tau using transgenic animals (Attems et al., 2011). The (P301L) Tau transgenic mice are a model system for the Tau-pathological aspects in AD in the absence of Aβ plaques. Interestingly, overexpression of this mutant (pathological form of) Tau protein significantly reduced Secretagogin expression in the brains of these mice.

As Secretagogin has been shown to be involved in insulin release from pancreatic cells, a possible link between Secretagogin and insulin in the brain can be imagined. Insulin together with insulin growth factor-1 (IGF-1) belongs to the main growth factors *in situ*, and therefore it is also added to the medium for neuronal cell culturing *in vitro*. The downstream signaling cascades are relatively well known. On the other hand, Secretagogin not only influences insulin synthesis in and secretion from endocrine cells, but it might also play a role in energy household and metabolism. Here we show that Secretagogin expression is increased in response to insulin *in vitro* supporting the idea that levels of Secretagogin could be regulated by circulating insulin or via different factors influencing insulin signaling pathways in the brain under normal and pathological conditions.

In this study we provide new insights into the expression patterns of Secretagogin in rodent’s (rat) brain that should help to formulate new hypotheses concerning the role of this CBP in the nervous system. Neuronal cell culture systems have meanwhile undergone further technical advancement and protocols are available for many more cell types of the CNS compared to the recent past. These primary neurons *in vitro* can be simple model systems to study the cellular functions and dynamics of Secretagogin. A high translational relevance of further knowledge on this protein is already apparent.

## Conflict of Interest Statement

The authors declare that the research was conducted in the absence of any commercial or financial relationships that could be construed as a potential conflict of interest.

## Supplementary Material

The Supplementary Material for this article can be found online at http://www.frontiersin.org/Molecular_Neuroscience/10.3389/fnmol.2012.00084/abstract

Supplementary Movie S1 **Three-dimensional reconstruction of a hippocampal interneuron co-expressing Parvalbumin and Secretagogin**. Rat brain slices were immunostained for Secretagogin (green) and Parvalbumin (red). Z-stacks were taken on a Leica confocal microscope from a Parvalbumin-positive interneuron in the CA1 region of the hippocampus co-expressing Secretagogin. The different subcellular distribution of both CBPs becomes clearly visible.

Supplementary Movie S2 **Subcellular association of Secretagogin with cis-Golgi marker GM130**. Rat brain slices were immunostained for Secretagogin (green) and GM130 (red). Z-stacks were taken on a Leica confocal microscope and recombined in a three-dimensional reconstruction of the cell. A non-random spatial proximity of compartment- or subcellular structure-associated Secretagogin and GM130 becomes evident.

## Abbreviations

CBP, calcium binding protein; nCBP, neuronal calcium binding protein; NCS, neuronal calcium sensor; SCGN, Secretagogin (human); Scgn, Secretagogin (rat).
